# Antimicrobial peptides secreted by equine mesenchymal stromal cells inhibit the growth of bacteria commonly found in skin wounds

**DOI:** 10.1186/s13287-017-0610-6

**Published:** 2017-07-04

**Authors:** Rebecca M. Harman, Steven Yang, Megan K. He, Gerlinde R. Van de Walle

**Affiliations:** Baker Institute for Animal Health, College of Veterinary Medicine, Cornell University, Ithaca, NY 14850 USA

**Keywords:** Equine mesenchymal stromal cells, Antimicrobial peptides

## Abstract

**Background:**

The prevalence of chronic skin wounds in humans is high, and treatment is often complicated by the presence of pathogenic bacteria. Therefore, safe and innovative treatments to reduce the bacterial load in cutaneous wounds are needed. Mesenchymal stromal cells (MSC) are known to provide paracrine signals that act on resident skin cells to promote wound healing, but their potential antibacterial activities are not well described. The present study was designed to examine the antibacterial properties of MSC from horses, as this animal model offers a readily translatable model for MSC therapies in humans. Specifically, we aimed to (i) evaluate the in vitro effects of equine MSC on the growth of representative gram-negative and gram-positive bacterial species commonly found in skin wounds and (ii) define the mechanisms by which MSC inhibit bacterial growth.

**Methods:**

MSC were isolated from the peripheral blood of healthy horses. Gram-negative *E. coli* and gram-positive *S. aureus* were cultured in the presence of MSC and MSC conditioned medium (CM), containing all factors secreted by MSC. Bacterial growth was measured by plating bacteria and counting viable colonies or by reading the absorbance of bacterial cultures. Bacterial membrane damage was detected by incorporation of N-phenyl-1-naphthylamine (NPN). Antimicrobial peptide (AMP) gene and protein expression by equine MSC were determined by RT-PCR and Western blot analysis, respectively. Blocking of AMP activity of MSC CM was achieved using AMP-specific antibodies.

**Results:**

We found that equine MSC and MSC CM inhibit the growth of *E. coli* and *S. aureus*, and that MSC CM depolarizes the cell membranes of these bacteria. In addition, we found that equine MSC CM contains AMPs, and blocking these AMPs with antibodies reduces the effects of MSC CM on bacteria.

**Conclusions:**

Our results demonstrate that equine MSC inhibit bacterial growth and secrete factors that compromise the membrane integrity of bacteria commonly found in skin wounds. We also identified four specific AMPs produced by equine MSC. The secretion of AMPs may contribute to the value of MSC as a therapy for cutaneous wounds in both horses and humans.

## Background

Chronic cutaneous wounds are a rapidly growing health care burden in human medicine. In the United States, chronic leg and foot ulcers alone affect 2.4–4.5 million people [1, 2]. Chronic skin wounds are defined as wounds that do not improve after 4 weeks or do not heal within 8 weeks [3]. Chronic wounds are classified into different categories and are caused by a variety of insults. A common feature of chronic wounds, regardless of initial cause, is colonization by pathogenic bacteria, which leads to an inflammatory host response and delayed wound healing [4, 5]. Chronic wounds typically contain a diversity of bacterial species that may interact to form matrices on wound surfaces called biofilms. Biofilms are particularly problematic as they show greater resistance to traditional antibiotics (Abx) compared to planktonic cells of the same species [6].

Antibiotic-resistant organisms are a challenge for the field of medicine in general, and for health care workers caring for patients with chronic cutaneous wounds in particular [7]. Alternatives to conventional Abx are currently being explored, with a focus on finding compounds that can kill bacteria directly rather than by disrupting metabolic activity and proliferation. Bacteria can survive insults to metabolic and proliferative pathways and evolve to avoid them, but they are less likely to become resistant to compounds that kill them directly [7, 8]. Naturally occurring or synthetic antimicrobial peptides (AMPs) could be used for the design of new classes of Abx [9–11]. The diverse array of AMPs acts through different mechanism, and because many AMPs are bactericidal as opposed to bacteriostatic, it is unlikely that bacteria will be able to respond to these AMPs by adopting resistance strategies [12–14]. This makes AMPs a promising class of molecules to be explored as novel antimicrobial therapies. A disadvantage of synthetic AMPs as an alternative to conventional Abx, however, is that they are costly to generate and purify [15]. Therefore, naturally occurring AMPs for therapeutic use may be a more practical and cost-effective substitute for traditional antibiotic therapy.

To explore this, a suitable animal model that allows for proper testing of the efficacy of naturally occurring AMPs to kill bacteria in chronic wounds of humans is warranted. Like humans, horses suffer from naturally occurring chronic wounds [16, 17] and bacterial infection of horse wounds delays the normal healing process by prolonging inflammation, reducing resident skin cell migration, and disrupting extracellular matrix formation [5, 18].

Mesenchymal stromal cells (MSC) are adult multipotent progenitor cells, present in a variety of tissues and organs [19] and contribute to healing processes by participating in the inflammatory, proliferative and remodeling phases of tissue repair [20–22]. Recent data show that paracrine signaling is the primary mechanism by which MSC contribute to tissue repair [23, 24]. Our laboratory has previously demonstrated that equine MSC conditioned medium (CM), comprising all factors secreted by MSC, can increase equine dermal fibroblast migration and block the effects of transforming growth factor beta-1 on equine dermal fibroblasts, providing the rationale for their potential in cutaneous wound management [25, 26]. The potential of equine MSC to contribute to wound healing by reducing bacterial loads has not been explored to date, although this seems achievable based on previous reports showing that human MSC possess antimicrobial properties such as killing *Escherichia coli* (*E. coli*) in rodent lung infection models, as well as reducing overall bacterial loads in septic mice [27–29].

Therefore, the aims of the present study were to (i) evaluate the in vitro effects of equine MSC on the growth of representative gram-negative and gram-positive bacterial species commonly found in skin wounds and (ii) define the mechanisms by which MSC inhibit bacterial growth. Our notable findings were that equine MSC and MSC CM can inhibit the growth of *E. coli* and *Staphylococcus aureus* (*S. aureus*) and that MSC CM can depolarize the cell membranes of these bacteria. In addition, we identified four AMPs produced by MSC, and observed that blocking these AMPs in MSC CM with antibodies reduces the effects of MSC CM on bacteria.

## Methods

### Cells

Equine MSC were isolated from the peripheral blood of three healthy warmblood mares between 8 and 12 years old, exactly as described previously [30]. The blood collection was approved by the Cornell Institutional Animal Care and Use Committee (IACUC # 2014-0038). Cells were seeded at a density of 1.6 × 10^5^ cells/cm^2^ in a T75 flask in culture medium, consisting of low glucose Dulbecco’s modified Eagle medium (DMEM) (Life Technologies, Grand Island, NY, USA) supplemented with 30% fetal bovine serum (FBS) (Atlanta Biological, Flowery Branch, GA, USA), 10^-7^ M low dexamethasone (Sigma-Aldrich, St. Louis, MO, USA), 50 μg/ml gentamycin, 1 × penicillin-streptomycin (P/S), and 2 mM L-glutamine (all from Life Technologies). Cultures were maintained at 37 °C with 5% CO_2_. At 70% confluency, cells were removed from flasks using 0.25% trypsin–EDTA and further cultured in expansion medium, which is identical to the culture medium but without dexamethasone. Equine MSC were characterized by immunophenotypical protein profiling using flow cytometry and their potential for trilineage differentiation, exactly as described previously [31].

The equine dermal fibroblast line NBL6 (ATCC, Manassas, VA, USA) was cultured in standard medium, consisting of DMEM supplemented with 10% FBS and 1× P/S, and maintained at 37 °C with 5% CO_2_.

### Bacterial cultures

*E. coli* 10536 and *S. aureus* 25923 (ATCC) colonies were maintained on Luria-Bertani (LB) agar (Life Technologies) plates at 4 °C for up to 1 month. For each experiment, a colony of the appropriate species was picked and used to inoculate 4 ml LB broth (Life Technologies), which was incubated on a shaker at 200 rpm, overnight at 37 °C, in a warm room with ambient air. Overnight cultures were diluted 1:100 in 4 ml LB broth and allowed to incubate, shaking at 200 rpm, at 37 °C until cultures reached the exponential growth phase, as determined by the absorbance reading of 1 ml culture at 600 nm using an Ultraspec 2100 pro spectrophotometer (Amersham Pharmacia Biotech, Cambridge, UK). Bacteria in the exponential growth phase were used for all experiments, unless stated otherwise.

### MSC-bacterial co-cultures

For experiments in which MSC and bacteria were co-cultured in direct contact with each other, 150,000 MSC or control NBL6 cells were plated per well in six-well plates in expansion or standard culture medium, respectively. After 24 hours (h), culture medium was removed, cell monolayers were rinsed twice with phosphate-buffered saline (PBS) and 1 ml DMEM was added to wells. Bacteria were added at 1.5 × 10^6^ per well. Control cultures contained bacteria in plain DMEM or DMEM with 2 × P/S without eukaryotic cells. All cultures were incubated for 6 h at 37 °C in a warm room with ambient air, while shaking at 100 rpm. The pH of the culture medium was measured at the start and end of the incubation period, and remained constant at a pH of 7.5 throughout the experiments. Culture media and cell monolayers, lysed with 1% saponin (Sigma-Aldrich) in distilled water, from each well were transferred to 5 ml tubes, vortexed to evenly distribute bacteria, and subsequently diluted in tenfold dilutions ranging from 1:10 to 1:1,000,000. Three 10 μl drops of each dilution were spotted on LB agar plates and allowed to incubate overnight at 37 °C. Bacterial colonies were counted and colony-forming units (CFU) per ml were calculated for each treatment.

Transwell experiments were carried out using the same numbers of cells and bacteria as were used for the direct contact co-cultures. For these assays, MSC or NBL6 cells were plated in 0.4 μm transwell inserts (Corning, Oneonta, NY, USA) fitted in six-well culture plates. Bacteria were added to lower chamber and, after incubation for 6 h at 37 °C while shaking at 100 rpm, in a warm room with ambient air, culture medium from the lower chamber was collected for assessment of live bacteria, as described above.

### Conditioned medium (CM) collection and treatments

CM was collected from MSC and NBL6 cells after 2 days of culture, when cells were 70% confluent. To this end, 6 × 10^5^ cells were seeded in a T75 flask with expansion medium. After 24 h, medium was removed, cell monolayers were rinsed twice with PBS, and 8 ml DMEM were added. Medium was collected 24 h later, centrifuged twice for 7 min at 300 × g to remove cellular debris, and used for subsequent experiments.

Experiments were also performed with equine MSC CM that was treated as follows: to inactivate large secreted proteins, CM was heat inactivated at 80 °C for 30 min or treated with 1 U/ml proteinase K (Qiagen, Valencia, CA, USA) for 6 h at 37 °C before use. To determine if the active factors responsible for the antibacterial effects of MSC CM are biologically stable, CM was frozen and thawed, or lyophilized and reconstituted before being used in assays. To determine the active subfraction of the CM responsible for inhibiting bacterial growth, CM was filtered using Amicon Ultra-15 centrifugal filters (EMD Millipore, Darmstadt, Germany), as per manufacturer’s instructions, and individual fractions containing secreted factors of specific molecular weights were used for subsequent experiments. To confirm the bioactive roles of identified AMP, CM was incubated with primary rabbit monoclonal antibodies against cystatin C (clone EPR4413) or rabbit polyclonal antibodies against elafin, lipocalin 2, cathelicidin (Abcam, Cambridge, MA, USA), combined equally for a final concentration of 4 μg/ml, for 1 h prior to the start of the experiments. CM incubated with 4 μg/ml rabbit IgG (Abcam) was used as a control.

### CM-bacterial co-cultures

CM collected as described above, was diluted 1:2 in LB broth for a total volume of 200 μl, and plated in triplicate wells of 96-well plates. Five hundred bacteria were added per well. Plates were incubated shaking at 100 rpm at 37 °C, in a warm room with ambient air, for 8 h and 16 h for *E. coli* and *S. aureus*, respectively. Wells containing DMEM diluted 1:2 in LB (negative control) and DMEM diluted 1:2 in LB with 2 × P/S (positive control) were also included. For the first experiment, the absorbance of the cultures was read using a 96-well Multiskan EX plate reader (Thermo Fisher Scientific, Waltham, MA, USA) at 600 nm and relative bacterial growth was calculated by comparing the absorbance of the CM wells or antibiotic-positive control wells to the absorbance of the DMEM-negative control wells. In addition, medium from each well was transferred to 1.5 ml tubes, vortexed to evenly distribute bacteria, and subsequently diluted in tenfold dilutions, spotted on LB agar plates and allowed to incubate overnight at 37 °C as described above. Bacterial colonies were counted and CFU per ml were calculated for each treatment. By comparing the two read-outs (CFU and absorbance), we determined that absorbance adequately reflected the CFU results, and used it as the read-out for subsequent CM-bacterial co-culture assays.

A subset of CM co-culture experiments was done using bacterial cultures in the post-exponential, stationary, growth phase CM. To this end, stationary bacterial cultures were washed with PBS, and bacterial pellets containing 1 × 10^9^ CFU *E. coli* or 1 × 10^8^ CFU *S. aureus* were resuspended in either 12.5 mg/ml polymixin B, 50 mg/ml nisin, or MSC CM. After 1 h at 4 °C, bacteria were rinsed twice with PBS by centrifugation, diluted in tenfold dilutions in PBS, spotted on LB agar plates and allowed to incubate overnight at 37 °C, as described above. Bacterial colonies were counted and CFU per ml were calculated for each condition.

### Biofilm assays

Biofilm assays were carried out based on a method described by O’Toole [32]. Briefly, 50 μl of equine MSC and NBL6 cell CM was pipetted into triplicate wells of u-bottom microtiter plates. DMEM and DMEM with 2 × P/S were included as negative and positive controls, respectively. An equal volume of bacteria from cultures in the exponential growth phase was added to each well, and plates were incubated for 72 h to allow for biofilm formation. *E. coli* biofilms were grown at room temperature, *S. aureus* biofilms were grown at 37 °C, in a warm room with ambient air. At 24 and 48 h, 50 μl CM or control medium was added to appropriate wells. After 72 h, medium was removed from wells and wells were rinsed 2 × with distilled water. *E. coli* biofilms were stained with 2.5% safranin and *S. aureus* biofilms were stained with 0.1% crystal violet for 10 min, after which stains were removed by rinsing biofilms twice with distilled water and allowed to air dry. Dye retained in biofilms was then solubilized using 30% acetic acid, and transferred to wells of 96-well flat bottom microtiter plates. Absorbance of solubilized dye was measured at 550 nm on an Infinite 200 pro plate reader (Tecan, Morrisville, NC, USA).

### Bacterial membrane depolarization assays

In brief, CM was added to an equal volume of DMEM in the first column of wells in a 96-well microtiter plate and titered in 1:2 dilutions in DMEM. One-N-phenylaphthylamine (NPN) (Sigma-Aldrich) was added to each well for a final concentration of 10 μm, and 50 μl of bacteria from cultures in the exponential growth phase were added. Plates were analyzed immediately in an Infinite 200 pro plate reader (Tecan) using an excitation of 355 nm and emission of 444 nm.

### Antibody arrays

A human proteome profiler antibody array, previously shown to cross-react with horse proteins [31, 33] was used, as per manufacturer’s instructions (R&D Systems, Minneapolis, MN, USA), to screen CM from equine MSC and equine dermal fibroblasts for the presence of AMPs. Positive signals were visualized using the ChemiDoc MP Imaging system (Bio-Rad, Hercules, CA, USA), normalized to the background, and data were quantified by measuring the sum of the intensities of the pixels within the spot boundary pixel area using image analysis software (Image Laboratory 4.1; Bio-Rad).

### Reverse transcription-polymerase chain reaction (RT-PCR)

RNA was extracted from cells using an RNeasy Mini Plus kit (Qiagen) and cDNA was synthesized using M-MLV Reverse Transcriptase (USB, Cleveland, OH, USA), per manufacturer’s protocols. Primers were designed using Primer3 software, based on sequences found in the National Center of Biotechnology Information (NCBI) GenBank and, where possible, primer sets spanned an intronic region to prevent amplification of genomic DNA (Table 1). RT-PCR using Taq DNA Polymerase (Life Technologies) was performed to amplify the AMP genes cystatin C (*CST3*), elafin (*PI3*), lipocalin 2 (*LCN2*), cathelicidin (*CAMP*) and beta defensin 2 (*DEFB4A*). Beta-2-microglobulin (*β2M*) was included as a reference gene. PCR products were run on a 1.5% agarose gel containing GelRed intercalating dye (Thermo Fisher Scientific) at 97 V for 1 h and gels were imaged on a Bio-Rad ChemiDoc MP system (Bio-Rad). Band intensities were measured using Bio-Rad Image Lab software and the intensities of the AMP gene bands were divided by the intensity of reference gene bands, to calculate relative band intensities.

**Table 1:** Primers used for RT-PCR

Gene product	Abbreviation	Forward primer (5′-3′)	Reverse primer (5′-3′)
Cystatin C	*CST3*	TTTCCTGTCACCGTACAGC	GCACAATGTCCGTGGTGAA
Elafin	*PI3*	GAGAAGGCTGAGTGCCAGAG	ACCAGCGAAATCATCTCCAG
Lipocalin 2	*LCN2*	TCAAGGATGACCAGTTCCAG	CCTTCCTGAAGAACACGATG
Cathelicidin	*CAMP*	GGGTAGATGGTCACTGTTGC	AGCCCATTCTCCTTGAAGTC
Beta defensin 2	*DEFB4A*	CGTTCCTCGTTGTCTTCCT	CACAGGTGCCAATCTGTTTC
Beta-2-microglobulin	*B2M*	GGGCTACTCTCCCTGACTGG	TACCTGCCCACACAGGTCAA

### Western blot and immunocytochemistry (ICC) analyses

Western blot analyses were performed, exactly as previously described [26], using the same Ab that were used to block AMP activity in MSC CM, diluted 1:500, followed by horseradish peroxidase (HRP)-conjugated goat anti rabbit secondary Ab (Jackson ImmunoResearch Labs, West Grove, PA, USA), diluted 1:20,000.

ICC was performed, exactly as previously described [34], using the same Ab that were used to block AMP activity in MSC CM, diluted 1:100, followed by HRP-conjugated goat anti rabbit secondary Ab, diluted 1:100.

### Statistical analyses

The Student’s *t* test for unpaired data was used to test for statistically significant differences in relative bacterial growth (filtration experiments), and relative band intensities (RT-PCR and Western blot analyses). When multiple *t* tests were performed on a single response variable within an experiment, the Holm-Šídák method was used to counteract the problems associated with multiple comparisons. One-way ANOVA, followed by the Tukey’s multiple comparison test was used to determine statistically significant differences in bacterial viability (co-culture experiments, CM experiments and bactericidal versus bacteriostatic effects of MSC CM experiments), relative growth (biofilm, CM experiments excluding filtration experiments, antibody-blocking experiments and NPN experiments, excluding filtration experiments. GraphPad software was used for analysis (GraphPad Software, La Jolla, CA, USA). Data given are the mean of three replicates and the bars show standard deviations.

## Results

### Equine mesenchymal stromal cells (MSC) inhibit bacterial growth via paracrine signaling

To begin assessing the antimicrobial potential of equine MSC, we designed in vitro experiments to determine if MSC can inhibit the growth of representative gram-positive and gram-negative bacteria, *E. coli* and *S. aureus,* respectively, that are commonly found in cutaneous wounds. Plain medium (DMEM) and antibiotics (Abx) were included as negative and positive controls, respectively, and experiments were also performed with the equine dermal fibroblast cell line NBL6, since skin fibroblasts are known to secrete antimicrobial compounds [35–37].

**Fig. 1:**
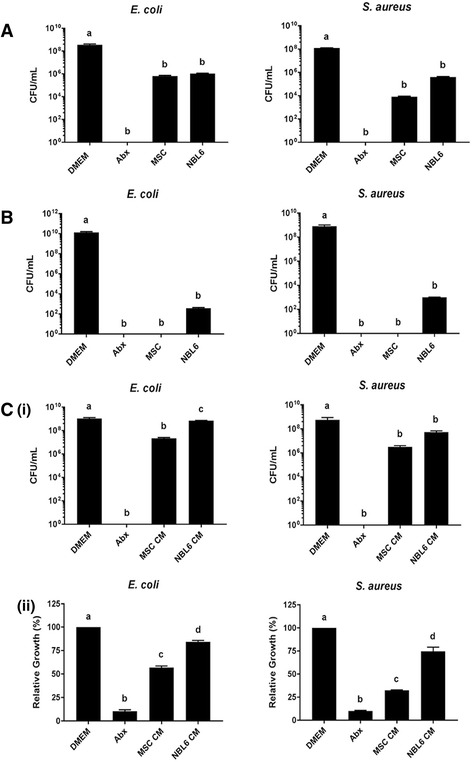
Equine MSC inhibit bacterial growth. **a** CFU per ml of *E. coli* (*left panel*) and *S. aureus* (*right panel*) following culture with DMEM, DMEM with Abx, MSC or NBL6 cells. **b** CFU per ml of *E. coli* (*left panel*) and *S. aureus* (*right panel*) following culture with DMEM, DMEM with Abx, MSC or NBL6 cells using transwell inserts. **c** CFU per ml (i) and relative growth based on absorbance readings at 600 nm (ii) of *E. coli* (*left panels*) and *S. aureus* (*right panels*). Cultures were grown in DMEM, DMEM with Abx, MSC CM or NBL6 CM each diluted 1:2 in LB broth. Different letters indicate statistically significant (*P* < 0.05) differences. *Abx* antibiotics, *CFU* colony-forming units, *CM* conditioned medium, *DMEM* Dulbecco’s modified Eagle medium, *MSC* mesenchymal stromal cells

When culturing bacteria in direct contact with equine MSC for 6 hours, like experiments previously carried out by Sung et al*.* with human MSC [38], we found that the growth of both bacterial species was significantly inhibited when compared to DMEM (Fig. 1a). Based on these encouraging results, we repeated these experiments with equine MSC cultured in transwell inserts to determine if the observed inhibition of bacterial growth was dependent on direct cell-to-cell contact. Equine MSC cultured in transwell inserts were still capable of effectively inhibiting the growth of bacteria within the time frame of the experiment (Fig. 1b), indicating that the observed effects are at least in part mediated by factors secreted by equine MSC, similar to what has been reported for human MSC [27, 38]. To determine whether equine MSC constitutively secrete factors with antimicrobial properties or if secretion is induced upon bacterial sensing, we cultured bacteria in CM collected from MSC and found that the relative bacterial growth, as assessed by CFU after 8 h for *E. coli*, and after 16 h for *S. aureus,* was significantly inhibited in the presence of MSC CM when compared to DMEM (Fig. 1c(i)). These results were directly compared to an alternative read-out for bacterial growth inhibition, namely the measurement of absorbance of bacterial cultures at 600 nm using a spectrophotometer. Although these two read-outs are not 100% similar (CFU/ml detects only live bacteria within a culture, while absorbance detects all bacteria that grew in the culture regardless of their viability at the time of measurement), the results obtained with absorbance reading reflected the results obtained by calculating CFU/ml (Fig. 1c(ii)). Since absorbance measurements are less time-consuming and allowed us to perform more technical replicates within each experiment, we decided to use absorbance as a read-out for subsequent CM experiments.

When comparing the results obtained with the equine MSC to the results obtained with the positive antibiotic (Abx) control and the NBL6 cells, we found that the levels of bacterial growth inhibition were not statistically different from the positive antibiotic control or NBL6 cells when we used CFU/ml as a read-out, except that fewer CFU/ml *E. coli* were detected when cultured in MSC CM as compared to NBL6 CM (Fig. 1a–c). When using relative growth as a read-out, the results suggest that the levels of bacterial growth inhibition caused by equine MSC CM are situated between the levels of inhibition obtained by Abx and NBL6 cells (Fig. 1c). This may reflect the mechanism by which MSC-secreted factors inhibit bacterial growth.

Taken together, these results show that equine MSC secrete factors that effectively inhibit the growth of both *E. coli* and *S. aureus*, to levels comparable or even greater than those observed with Abx or dermal fibroblasts.

### Equine MSC secrete factors that inhibit bacterial biofilm formation

**Fig. 2:**
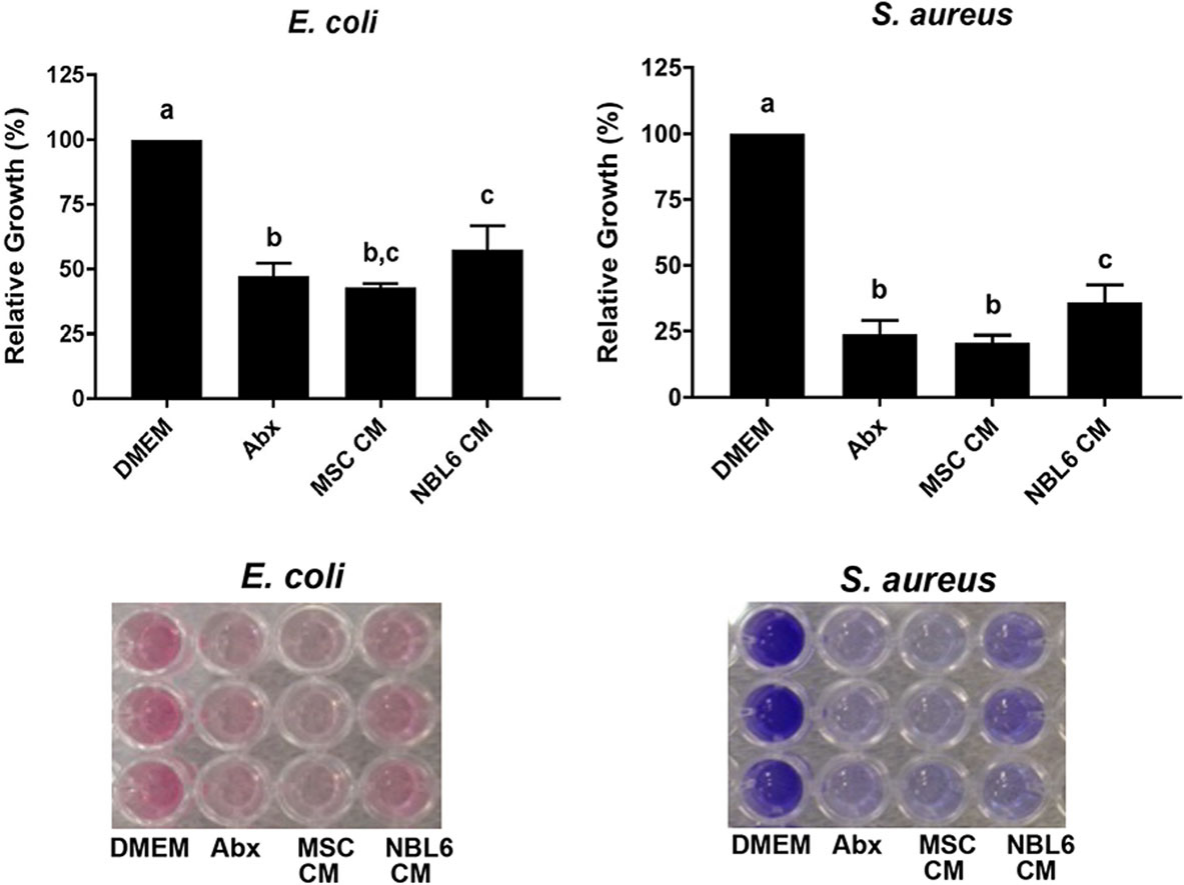
Equine MSC constitutively secrete factors that inhibit bacterial biofilm formation. Relative growth of *E. coli* (*left panel*) and *S. aureus* (*right panel*) biofilms grown in DMEM, DMEM with Abx, MSC CM or NBL6 CM. Representative images of dye taken up by biofilms are shown below graphs. Different letters indicate statistically significant (*P* < 0.05) differences. n = 3. *Abx* antibiotics, *CM* conditioned medium, *DMEM* Dulbecco’s modified Eagle medium, *MSC* mesenchymal stromal cells

Because biofilms contribute to the inhibition of cutaneous wound healing [7, 39] we assessed the effect of equine MSC CM on biofilm formation using an in vitro biofilm assay established by O’Toole [32]. Again, equine MSC CM significantly reduced the growth of both *E. coli* and *S. aureus* biofilms, to an extent that was virtually identical to that observed with the antibiotic control for both types of bacteria, and to levels that differed significantly from NBL6 CM for *S. aureus* (Fig. 2).

### Equine MSC secrete stable, low molecular weight molecules that inhibit bacterial growth

**Fig. 3:**
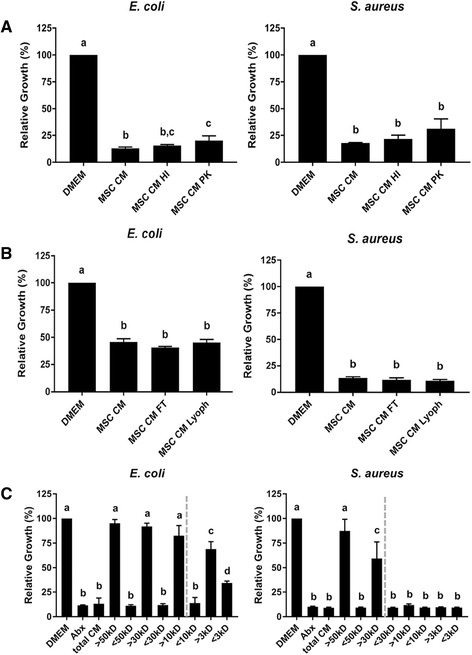
Equine MSC secrete stable, low molecular weight factors that inhibit bacterial growth. **a** Relative growth of *E. coli* (*left panel*) and *S. aureus* (*right panel*) based on absorbance readings at 600 nm. Cultures were grown in DMEM, MSC CM, heat-inactivated MSC CM (*HI*) or proteinase K-treated CM (*PK*) each diluted 1:2 in LB broth. **b** Relative growth of *E. coli* (*left panel*) and *S. aureus* (*right panel*) based on absorbance readings at 600 nm. Cultures were grown in DMEM, MSC CM, frozen-thawed MSC CM (*FT*) or lyophilized-reconstituted CM (*Lyoph*) each diluted 1:2 in LB broth. **c** Relative growth of *E. coli* (*left panel*) and *S. aureus* (*right panel*) based on absorbance readings at 600 nm. Cultures were grown in DMEM, MSC CM, and MSC CM fractioned by size of secreted factors. Different letters indicate statistically significant (*P* < 0.05) differences. n = 3. *Abx* antibiotics, *CM* conditioned medium, *DMEM* Dulbecco’s modified Eagle medium, *MSC* mesenchymal stromal cells

To determine the characteristics of the bioactive factors with antimicrobial properties secreted by equine MSC, the following set of experiments was performed. First, we used boiling (heat inactivation (HI)) and proteinase K (PK) treatment to inactive large proteins in the MSC CM, and compared the levels of bacterial growth inhibition to those obtained with untreated MSC CM. We found that HI CM still significantly inhibited the growth of both types of bacteria when compared to medium control, more specifically to levels indistinguishable from those obtained with the same MSC CM that was left untreated (Fig. 3a). Similar results were observed for the PK-treated CM, although the growth inhibition of *E. coli* was not as robust when compared to the untreated CM (Fig. 3a). Next, we froze and thawed, as well as lyophilized and reconstituted, CM before its use in the bacterial assays in order to determine the stability of the active factors. We observed that neither freezing nor lyophilizing significantly altered the growth inhibitory effects of CM on either bacterial species tested, as compared to fresh CM (Fig. 3b). Finally, we filtered the MSC CM to obtain subfractions containing secreted factors of specific sizes, and found that secreted factors of less than 10 kDa and less than 30 kDa significantly inhibited the growth of *E. coli* and *S. aureus*, respectively (dotted lines, Fig. 3c).

### Equine MSC secrete factors that depolarize bacterial cell membranes, but do not appear to immediately kill bacteria

**Fig. 4:**
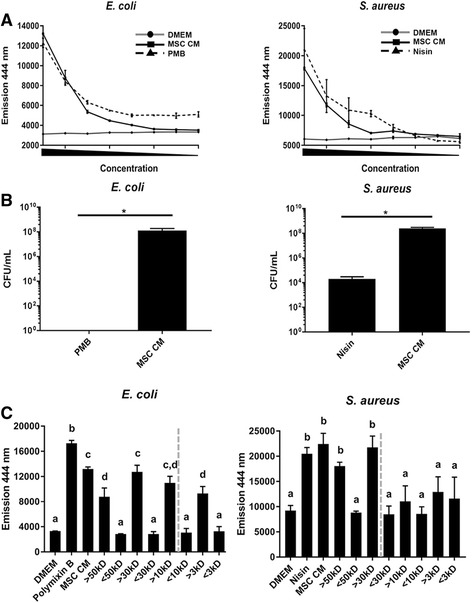
Equine MSC secrete factors that depolarize bacterial cell membranes, but do not cause immediate cell death. **a** Incorporation of NPN into depolarized *E. coli* membranes (*left panel*) and *S. aureus* membranes (*right panel*) treated with MSC CM compared to high concentrations of compounds known to depolarize bacterial cell membranes. **b** CFU per ml of *E. coli* (*left panel*) and *S. aureus* (*right panel*) following culture at 4 °C with bactericidal compounds polymixin B (*PMB*), nisin or MSC CM. *P < 0.05 **c** NPN incorporation by depolarized *E. coli* (*left panel*) and *S. aureus* (*right panel*) membranes as measured by fluorescent emission at 444 nm. Bacteria were treated with DMEM, MSC CM, and MSC CM fractioned by size of secreted factors. Different letters indicate statistically significant (*P* < 0.05) differences. n = 3. *CFU* colony-forming units, *CM* conditioned medium, *DMEM* Dulbecco’s modified Eagle medium, *MSC* mesenchymal stromal cells

In parallel, we decided to evaluate whether the observed MSC CM-mediated bacterial growth inhibition is caused by a membrane-depolarizing mechanism. To this end, we exposed bacteria to MSC CM in the presence of 1-N-phenylaphthylamine (NPN), a compound that is excluded by intact outer membranes of bacteria but taken up into the hydrophobic interior of outer membranes that have been depolarized. Consequently, a strong fluorescent NPN signal is correlated with disrupted bacterial membranes [40, 41]. The results from these experiments demonstrated that the equine MSC CM causes membrane depolarization of *E. coli* as well as *S. aureus* (i) in a concentration-dependent manner and (ii) to degrees similar to Abx known to depolarize gram-negative and gram-positive bacterial membranes; polymixin B and nisin, respectively (Fig. 4a). Since polymixin B and nisin are bactericidal by killing bacteria shortly after contact [42, 43], we decided to evaluate whether the observed equine MSC CM-mediated bacterial membrane damage resulted in similar immediate killing of bacteria. To this end, we directly compared the number of viable bacteria after CM treatment to the numbers obtained after exposure to these two bactericidal Abx. Based on the difference in number of bacteria between these two types of treatments, with both bactericidal Abx resulting in significantly lower CFU/ml (*P* < 0.05, Fig. 4b), we concluded that treatment of stationary, non-dividing bacteria with equine MSC CM did not immediately kill bacteria to the same extent as the bactericidal control compounds.

We then repeated the NPN experiments with the different CM subfractions and found that *E. coli* exposed to MSC-secreted factors greater than 10 kDa took up more NPN than *E. coli* exposed to MSC-secreted factors smaller than 10 kDa, indicating that the bioactive factors that depolarize *E. coli* membranes are most likely 10 kDa or greater in size (*dotted line*, Fig. 4c). Likewise, *S. aureus* exposed to MSC-secreted factors greater than 30 kDa took up more NPN than *S. aureus* exposed to MSC-secreted factors smaller than 30 kDa, indicating that the bioactive factors that depolarize *S. aureus* membranes are most likely 30 kDa or greater in size (dotted line, Fig. 3c).

Collectively, these data indicate that equine MSC secrete a variety of bioactive factors of different sizes that affect bacteria by various modes of action, including membrane depolarization.

### Equine MSC secrete antimicrobial peptides (AMPs)

**Fig. 5:**
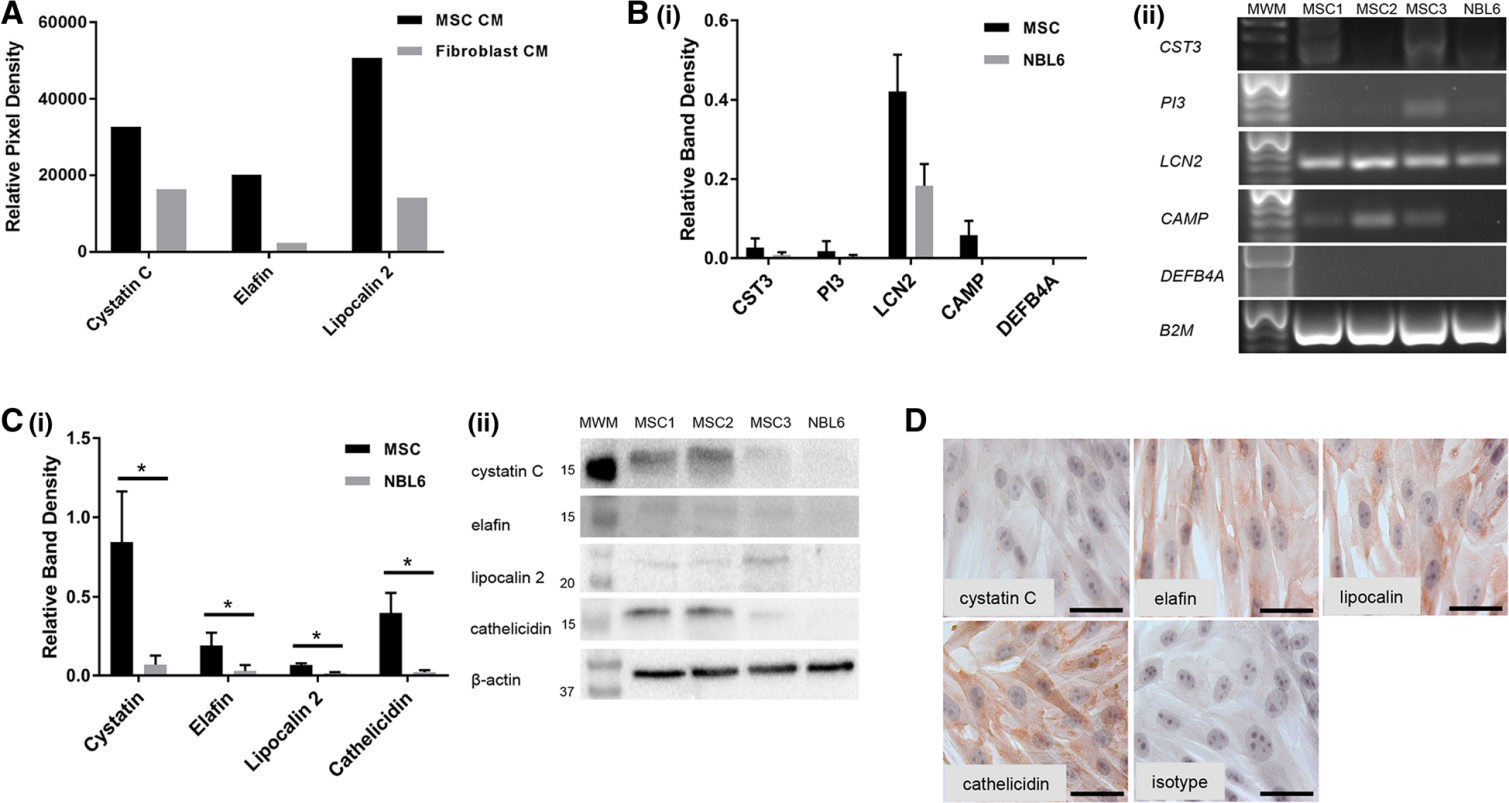
Equine MSC express at least four AMPs. **a** Detection of three AMPs in MSC CM and control CM as detected by a commercially available antibody array. n = 1. **b** (i) Relative band densities of five AMP transcripts in equine MSC and NBL6 compared to the reference transcript *B2M* as detected by RT-PCR. (ii) Representative images of PCR products run on agarose gels. Lane 1 = molecular weight marker; Lanes 2, 3, and 4 = equine MSC derived from three different horses; Lane 4 = NBL6. **c** (i) Relative band densities of four AMPs in equine MSC and NBL6 compared to the reference protein β-actin as detected by Western blot. (ii) Representative images of protein lysates run on polyacrylamide gels, transferred to PVDF membranes, and probed with anti-AMP antibodies. Lane 1 = molecular weight marker; Lanes 2, 3 and 4 = equine MSC derived from three different horses; Lane 4 = NBL6. **d** Images of equine MSC labeled with four anti-AMP antibodies (*red*). Scale bars = 10 μm, *P < 0.05, n = 3. *CM* conditioned medium, *MSC* mesenchymal stromal cells

Since AMPs are a class of low molecular weight molecules known to directly kill bacteria by forming pores in bacterial membranes [13, 14], we decided to evaluate the secretion of AMPs by equine MSC. We preliminarily screened the equine MSC CM, and a control dermal fibroblast CM for the presence of AMPs using a human proteome profiler array, which can detect the relative levels of over 100 proteins simultaneously (n = 1). The expression of three AMPs, namely cystatin C, elafin, and lipocalin 2, were readily detected in MSC CM, at higher levels than in the control CM (Fig. 5a). The expression of these AMPs, as well as the expression of cathelicidin and beta defensin 2 (the most common mammalian AMPs, both of which have previously been described in the horse [12, 44]), was then evaluated in the three equine MSC cultures that were used for the bacterial assays by RT-PCR using primers that had previously been confirmed by our laboratory to amplify products of the appropriate lengths in horse cells. Except for the gene *DEFB4A* (encoding beta defensin 2), expression of the AMP transcripts *CST3*, *PI1*, *LCN2*, and *CAMP* (encoding cystatin C, elafin, lipocalin and cathelicidin, respectively) could be detected (Fig. 5b). Next, Western blot analyses as well as immunocytochemistry (ICC) were performed to confirm protein expression of those AMPs that were detected on an mRNA level (Fig. 5c, d). For comparison, we also performed RT-PCR and Western blot analyses on NBL6 cells and found that whereas these cells showed similar expression levels of *CST3*, *PI1*, *LCN2*, and *CAMP* transcripts (Fig. 5b), the expression of the corresponding proteins cystatin C, elafin, lipocalin and cathelicidin, was significantly lower in NBL6 compared to MSC cultures (Fig. 5c). This nicely corresponded with the original preliminary screening of dermal fibroblast CM using the human proteome profiler array (Fig. 5a).

### The ability of equine MSC to inhibit bacterial growth and induce bacterial membrane damage is greatly reduced by pretreatment of CM with anti-AMP antibodies

**Fig. 6:**
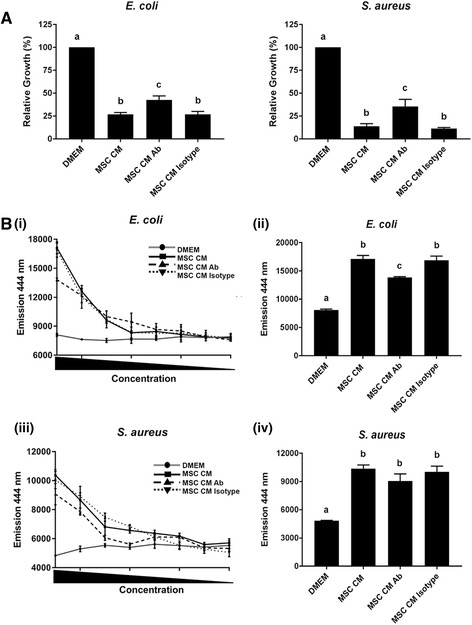
Blocking AMP activity decreases the effects of MSC CM on bacteria. **a** Relative growth of *E. coli* (*left panel*) and *S. aureus* (*right panel*) based on absorbance readings at 600 nm. Cultures were grown in DMEM, MSC CM, and MSC CM pre-incubated with anti-AMP antibodies (*Ab*) or MSC CM pre-incubated with an isotype control antibody, each diluted 1:2 in LB broth. **b** Incorporation of NPN into depolarized *E. coli* and *S. aureus* membranes in the presence of decreasing concentrations of DMEM, MSC CM, and MSC CM pre-incubated with anti-AMP antibodies or MSC CM pre-incubated with an isotype control antibody as measured by fluorescent emission at 444 nm (i and iii). Emission values from *E. coli* and *S. aureus* with treatments diluted 1:2 (ii and iv). Different letters indicate statistically significant (*P* < 0.05) differences. n = 3. *CFU* colony-forming units, *CM* conditioned medium, *DMEM* Dulbecco’s modified Eagle medium, *MSC* mesenchymal stromal cells

To provide a link between the AMPs detected in equine MSC and the observed antimicrobial effects of the MSC CM, we repeated the bacterial growth inhibition assays using MSC CM that was pretreated with antibodies against these AMPs. Although we found that anti-AMP antibody-pretreated CM still reduced bacterial growth significantly when compared to the DMEM control (Fig. 6a), the bacterial growth inhibition by pretreated CM was significantly lower compared to untreated MSC CM or CM pretreated with isotype control antibodies (Fig. 6a). This suggests that cystatin C, elafin, lipocalin and/or cathelicidin are involved in, but not solely responsible for, the MSC CM-mediated growth inhibition of *E. coli* and *S. aureus*. We then corroborated these results by repeating the NPN experiments using antibody-pretreated MSC CM. A significant difference in *E. coli* membrane depolarization was observed compared to the DMEM control that was significantly less pronounced than the effects of untreated MSC CM or CM pretreated with isotype control antibodies (Fig. 6b (i and ii)). Interestingly, no significant difference in the membrane depolarization of *S. aureus* was observed between the antibody-pretreated MSC CM and untreated MSC CM or CM pretreated with isotype control antibodies*,* indicating that MSC-secreted factors other than cystatin C, elafin, lipocalin or cathelicidin are responsible for the membrane damage detected in this bacterial species (Fig. 6b (iii and iv)).

Taken together, these results demonstrate that the equine MSC secreted AMPs cystatin C, elafin, lipocalin and cathelicidin, are, at least in part, responsible for the observed growth inhibition of *E. coli* and *S. aureus* cultured in MSC CM. In addition, these AMPs also contribute to the membrane damage detected in *E. coli*, but not *S. aureus*.

## Discussion

This study is the first to demonstrate that equine mesenchymal stromal cells (MSC) possess antibacterial properties by showing that MSC inhibit the growth of *E. coli* and *S. aureus*, and depolarize the membranes of these bacteria in vitro. Moreover, this study describes the presence of four distinct antimicrobial peptides (AMPs) in the equine MSC secretome, namely cystatin C, elafin, lipocalin 2, and cathelicidin, and demonstrates that these AMPs are at least partially responsible for the antimicrobial effects of MSC.

The MSC-produced AMPs identified in this study represent several classes of antimicrobial compounds, some of which have been documented to be produced by MSC from other sources and species. Cystatin C has a mode of action that has not been fully elucidated to date, but has been identified as a secreted product of both murine bone marrow-derived and human adipose tissue-derived MSC [45, 46]. Lipocalin 2 limits bacterial growth by sequestering iron-containing siderophores and has been identified in the secretome of mouse bone marrow-derived MSC [47]. Cathelicidin is a cationic a that kills bacteria directly by disrupting membrane polarization and produces a cleavage product (LL-37) that has previously been described as a secreted product of human bone marrow-derived MSC [27, 48]. Elafin is known to kill bacteria by disrupting the membrane integrity. To our knowledge, elafin has not been reported as a product of MSC, so this study is the first to report on the production of this AMP by MSC.

Besides these four AMPs, we also evaluated the presence of beta defensin 2 in equine MSC since this AMP (i) has been described to be secreted by human umbilical cord-derived MSC [38, 49] and (ii) belongs to a ubiquitous family of AMPs that are found in most mammals, including horses [44, 50]. However, and to our surprise, we could not detect beta defensin 2 in equine MSC. This was not due to an improperly functioning of the PCR primers, as we have used the same primers to confirm expression of this AMP transcript in equine keratinocytes (data not shown). To identify the complete spectrum of equine MSC-derived AMPs, we plan to use a more unbiased and global approach (e.g., liquid chromatography-mass spectrometry (LC-MS/MS)) in future experiments.

The bacterial growth inhibition and biofilm assays were run in parallel with an equine dermal fibroblast cell line, based on the knowledge that the skin of most mammals produces AMPs [50, 51] and AMP mRNA and proteins have been identified in horse skin [44]. As expected, we found that equine dermal fibroblasts inhibit bacterial growth and produce specific AMPs. To the best of our knowledge, the AMP production by horse skin fibroblasts had not been evaluated previously. Interestingly, the expression of AMPs by the horse dermal fibroblast cell line NBL6 was not equivalent to that of equine MSC, with equine MSC producing higher levels of cystatin C, elafin, lipocalin 2 and cathelicidin. This further emphasizes the therapeutic potential of equine MSC in cutaneous wound management, as the administration of MSC or MSC-secreted products could supplement AMPs expressed at low levels in the skin, therefore expanding the range of AMPs locally present to fight skin infections.

Although the results of this study clearly demonstrate that equine MSC have antimicrobial properties against both *E. coli* and *S. aureus*, there appears to be a difference in the underlying mechanisms targeting each species. Based on the CM fractionation experiments, we demonstrated that secreted factors less than 10 kDa in size are responsible for the observed growth inhibition of *E. coli* whereas secreted factors less than 30 kDa appear to inhibit the growth of *S. aureus*. Moreover, MSC-secreted factors greater than 10 kDa contributed to most membrane damage seen in *E. coli,* whereas secreted factors greater than 30 kDa caused membrane damage to *S. aureus.* And although in the current study, we did not fully investigate the exact underlying mechanisms by which equine MSC CM affects different bacteria species, our results do clearly show that MSC-secreted factors (i) inhibit bacterial growth and (ii) depolarize the outer membranes of bacteria. It will be interesting, therefore, to study the effects of MSC CM on additional bacterial species commonly found in equine skin wounds, such as the gram-negative bacteria *Pseudomonas aeruginosa* and *Acinetobacter baumannii*, and the gram-positive *Aerococcus viridians*, *Staphylococcus warneri*, and *Staphylococcus epidermidis* [39], to determine whether there are indeed distinct mechanisms used by equine MSC to target gram-positive versus gram-negative bacteria. As most of these bacterial species are also found in human skin wounds [52, 53], results will be relevant to human as well as veterinary medicine.

Since we found that equine MSC secrete a variety of AMPs that appear effective against both gram-positive and gram-negative bacteria, these cells may serve as a broad-spectrum treatment to control bacterial growth and kill bacteria, without leading to resistance. Work by other groups has shown that in addition to controlling bacteria, AMPs and other host defense peptides directly contribute to wound healing by inducing cell migration and proliferation, promoting angiogenesis, and in general, accelerating the healing process [54]. This dual function of peptides to promote wound healing, combined with the ease and low cost of isolating MSC and collecting CM, makes MSC CM an ideal biological source for naturally occurring peptides as well as other factors that promote wound healing. Our data indicating that equine MSC CM can be lyophilized and still retain anti-bacterial activity suggest that using MSC CM therapeutically may be practical as well, providing a stable, off-the-shelf product for clinical use.

Taken together, our group focuses on the potential of equine MSC to be used as a therapy for skin wounds and the data generated in this study suggests that in addition to positively affecting resident skin cells, as we have demonstrated previously [25, 26, 31], equine MSC may also improve cutaneous wound healing by reducing the bacterial load in wounds. Next, we intend to evaluate the efficacy of equine MSC in vivo by assessing how these cells promote skin wound healing and affect bacterial burden, in both acute and chronic wounds.

## Conclusions

Mesenchymal stromal cells (MSC) have been reported to provide paracrine signals that promote cutaneous wound healing, but (i) the effects of equine MSC on the growth of gram-negative and gram-positive bacterial species commonly found in skin wounds and (ii) the mechanisms by which equine MSC inhibit bacterial growth had not been explored thus far. The present study is the first to show that equine MSC possess antimicrobial properties by inhibiting the growth of *E. coli* and *S. aureus*, in part by secreting antimicrobial peptides (AMPs) and depolarizing bacterial cell membranes. This antibacterial activity may contribute to the value of MSC as a therapy for chronic cutaneous wounds in both horses and humans, where colonization by pathogenic bacteria commonly inhibits normal healing.

## Abbreviations

Abx: Antibiotics
AMP: Antimicrobial peptide
CAMP: Cathelicidin
CFU: Colony-forming units
CM: Conditioned medium
*CST3*: Cystatin C
*DEFB4A*: Beta defensin 2
DMEM: Dulbecco’s modified Eagle medium
FBS: Fetal bovine serum
HI: Heat inactivation
ICC: Immunocytochemistry
LB: Luria-Bertani
*LCN2*: Lipocalin 2
MSC: Mesenchymal stromal cells
NPN: 1-N-phenylaphthylamine
P/S: Penicillin-streptomycin
PBS: Phosphate-buffered saline
*PI3*: Elafin
PK: Proteinase K
RT-PCR: Reverse transcription-polymerase chain reaction
*β2M*: Beta-2-microglobulin
